# A study of different minimum segment area parameters on automatic IMRT plans for cervical cancer using Pinnacle^3^ 9.10 TPS: Retraction

**DOI:** 10.1097/MD.0000000000039829

**Published:** 2024-09-06

The article “A study of different minimum segment area parameters on automatic IMRT plans for cervical cancer using Pinnacle^3^ 9.10 TPS,”^[1]^ which published in Volume 101, Issue 36 of *Medicine*, is being retracted due to duplicate publication.^[2]^ All accepted manuscripts are subjected to a plagiarism check prior to being transmitted to production; however, the duplication was not detected due to it being published in another language. A concerned reader notified the Editorial Office of the duplicate publication, and a native speaker verified that the translated article largely shared the same objective, method, and results. The authors were notified of the retraction.
